# The Immunogenicity of OMP31 Peptides and Its Protection Against *Brucella melitensis* Infection in Mice

**DOI:** 10.1038/s41598-019-40084-w

**Published:** 2019-03-05

**Authors:** Fengbo Zhang, Zhiwei Li, Bin Jia, Yuejie Zhu, Pan Pang, Chuntao Zhang, Jianbing Ding

**Affiliations:** 1 Department of Clinical Laboratory, The First Affiliated Hospital of Xinjiang Medical University, Urumqi, 830011 Xinjiang China; 2 Department of Clinical Laboratory, The People Hospital of Xinjiang, Urumqi, 830001 Xinjiang China; 3 Reproductive Medicine Center, The First Affiliated Hospital of Xinjiang Medical University, Urumqi, 830011 Xinjiang P.R. China; 4 Department of Immunology, College of Basic Medicine of Xinjiang Medical University, Urumqi, 830011 Xinjiang China

## Abstract

Given brucellosis is a widespread zoonosis in the world, a safe and effective vaccine is urgently needed. Recent trend in vaccine design has shifted to epitope-based vaccines that are safe and specific. In this study, peptide containing both T-cell and B-cell epitopes of OMP31 was synthesized and used to immunize the mice by nasal administration. The protective efficacy was evaluated. Mice immunized with the B epitope or TB epitope peptides of OMP31 had higher levels of IgG1 and IgG2a in the serum. While the BALB/c mice immunized with peptides containing T cell epitope or TB epitope of OMP31 showed high degree of IFN-γ-producing T cells in the lymphocytes from the respiratory draining lymph nodes and spleen. After intranasally challenged with 5 × 10^5^ CFU of *Brucella melitensis* (strain 16 M), the bacterial loads in lung of the immunized mice were significantly lower than control group. These data demonstrate for the first time that peptides of OMP31 containing T epitope, B epitope or TB epitopes are of high immunogenicity and thus can protect host from *Brucella melitensis* infection in lung.

## Introduction

Brucellosis is a widespread zoonosis caused by *Brucella* species and can induce severe illness in humans and substantial economic losses in livestock^1–4^. *Brucella melitensis* (*B*. *melitensis*) are the main causative agents of Brucellosis in humans. The disease has a tendency towards chronicity and persistence, which can develop into a granulomatous disease capable of affecting any organ system. The timely and accurate diagnosis of human brucellosis continues to challenge clinicians because of its non-specific clinical features, slow growth rate in blood cultures, and the complexity of its sero-diagnosis.

Human brucellosis poses significant economic and health concerns in Mediterranean areas, the Middle East, South and Central America and Asia. There are more than 500,000 cases reported annually worldwide^5,6^. In livestock, brucellosis is responsible for reproductive loss resulting from abortion, birth of weak offspring, or infertility.

*Brucella* can cause the infection via the mucosal surface of the host. For livestock, the main route of exposure is by ingestion or inhalation of microorganisms. Infection in humans occurs mainly by consumption of contaminated milk and meat products, contact with fluids of infected animals and inhalation of infectious aerosols^7,8^. What is shared between animal and human *Brucella* transmission is the naso-oropharyngeal mucosa being impacted by *Brucella*, but not the intestinal mucosa. Hence, a vaccine aimed at inducing the respiratory system mucosal immune response has a high potential for success. Unfortunately, there is still no available human brucellosis vaccine. All commercially available animal vaccines are based on live attenuated strains of *Brucella* that can induce abortions in pregnant animals and are potentially infectious to humans^9–11^.

Owing to disadvantages of live attenuated vaccines, replacing these vaccines by peptide ones would be a great improvement for safety reasons. An ideal peptide vaccine should include multivalent B cell epitopes and T cell epitopes to elicit high humoral immunity and cell immunity response^12,13^. Peptide vaccines have been found to be effective in preventing diseases, such as Chlamydia trachomatis^14^, foot-and-mouth disease^15^ and *Mycobacterium tuberculosis*^16^. Several *Brucella* components have been used as vaccines against brucellosis. Previous study indicates that peptide vaccine designed based on *Brucella* components could provide protection against brucellosis^17^. The 31KDa outer membrane protein (Omp31) is a major membrane protein of *Brucella* and plays an important role in conferring the protection against *B*. *melitensis*^18–21^. Although Omp31 is of great potential as an antigen for vaccination^22–30^, it is also an important virulence factor of *Brucella* species. One alternative of Omp31 vaccine is to develop peptide vaccine, which can be immunized by nasal administration and provide protection in the respiratory tract.

In this study, we analyzed T cell and B cell epitopes of OMP31 by bioinformatics. Then we synthesized the corresponding epitopes and used them as vaccines to immunize mice. The effects of OMP31 peptide vaccines on the immune response and protection against *B*. *melitensis* infection were evaluated in mice model.

## Results

### Immunization with peptides containing B epitope and TB epitope induces specific systemic humoral immunity and mucosal immunity of respiratory tract

**Figure 1:**
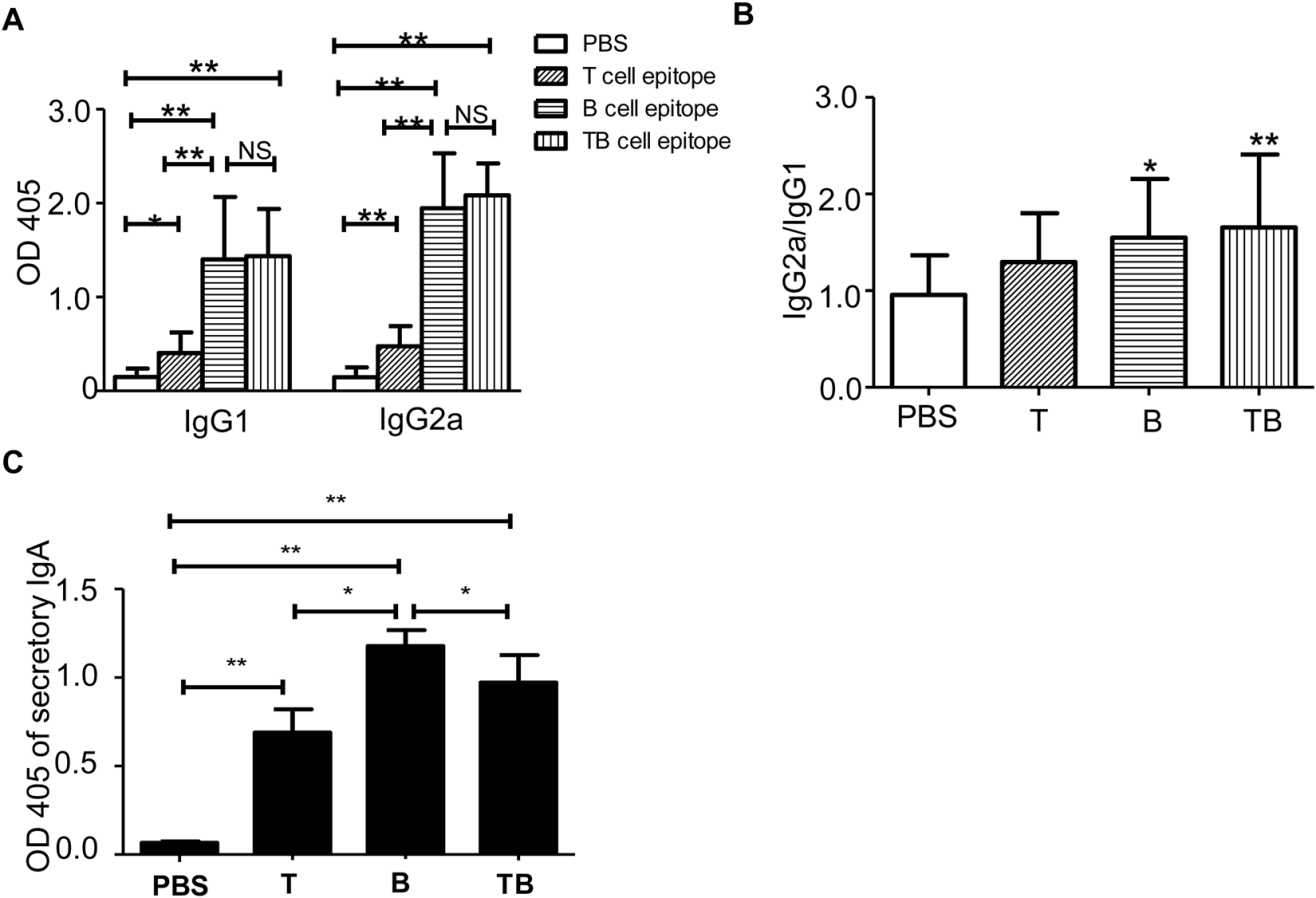
IgG1 and IgG2a levels in mice after peptides immunization. Mice were immunized with PBS or respective peptides. The specific IgG1 and IgG2a antibodies in mouse serum and sIgA in bronchoalveolar lavage fluid were detected by ELISA. (**A**) The specific IgG1, IgG2a antibodies in the immunized mice serum. (**B**) The IgG2a/IgG1 ratio. (**C**) The sIgA level in bronchoalveolar lavage fluid. Data are shown as mean ± SD. One-way ANOVA was used for analysis. **P < *0*.*01*, *P < *0*.*05*.

To verify whether peptides containing B epitope, T epitope or TB epitope could promote the humoral immune response, we immunized mice with different peptides, respectively. The serum was collected and the levels of specific IgGl and IgG2a antibodies were measured by ELISA. Both IgG1 and IgG2a in mice immunized with B peptide, T epitope or TB epitope were significantly higher than those of PBS control group (*P* < *0*.*01*) (Fig. 1A). To further explore the major T cell types in different peptides immunized mice, we compared the levels of IgG2a/IgG1 ratio. The results showed that IgG2a/IgG1 ratio in mice immunized with B peptide or TB epitope was significantly higher than that in PBS control group (*P* < *0*.*01*) and T epitope (*P* < *0*.*05*) (Fig. 1B), indicating that the T cell immune response is biased towards Th1 reaction in the B peptides and TB peptides immunized mice. To investigate whether the respiratory mucosal immune response is also activated by peptides containing B epitope, T epitope or TB epitope, we examined the level of secretory IgA (sIgA) in the bronchoalveolar lavage fluid. As shown in Fig. 1C, the sIgA level after immunization with peptides containing T epitope, B epitope and TB epitope was significantly higher (*P* < *0*.*05*). Additionally, the sIgA level in the mice immunized with peptides containing T epitope and TB epitope was significantly lower than that of the B epitope immunization group (*P* < *0*.*05*). These results indicate that peptides containing B epitope and TB epitope could promote specific systemic humoral immunity and mucosal immunity of respiratory tract.

### T epitope or TB epitope containing peptides induces specific T cell immunity

**Figure 2:**
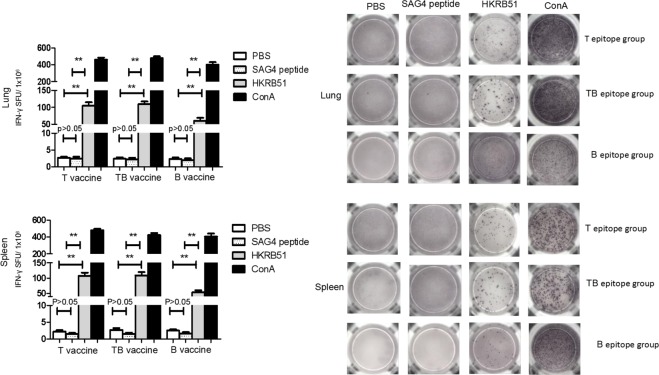
The specific T cells response by IFN-γ ELISPOT after peptides immunization. Thirty days after the last immunization in mice, the lung and splenocytes were isolated and stimulated with HKRB51 (Heat killed RB51). SAG4 peptide and PBS were used as negative controls. IFN-γ producing T cells were detected by ELISPOT assay. (**A**) Number of IFN-γ producing T cells. One-way ANOVA was used for the Statistical Analysis. Data are shown as mean ± SD. **P < *0*.*01*. *P < *0*.*05*. SFU, spot-forming units. (**B**) Representative ELISPOT results.

To analyze whether peptides containing T epitope, B epitope or TB epitope could activate the T cell immune response, the lymphocytes in the respiratory draining lymph nodes and spleen were isolated from the peptides immunized mice. And the specific T cell responses were tested by IFN-γ ELISPOT assay after stimulation with heat-killed RB51 (HKRB51) or ConA. PBS and SAG4 peptide were used as negative controls. As shown in Fig. 2A,B the IFN-γ secreting T cells of the respiratory lymph nodes and splenocytes in T peptides and TB peptides immunized mice were significantly higher than those of PBS group (*P* < *0*.*01*) or SAG4 peptide group (*P* < *0*.*01*). There was no significant difference between PBS group and SAG4 peptide group (*P > 0*.*05*). These data indicate that peptides containing T epitope or TB epitope can induce specific T cell immunity.

### Peptide immunization provides partial protect against *B*. *melitensis* infection in lung but cannot confer protection against its dissemination

**Figure 3:**
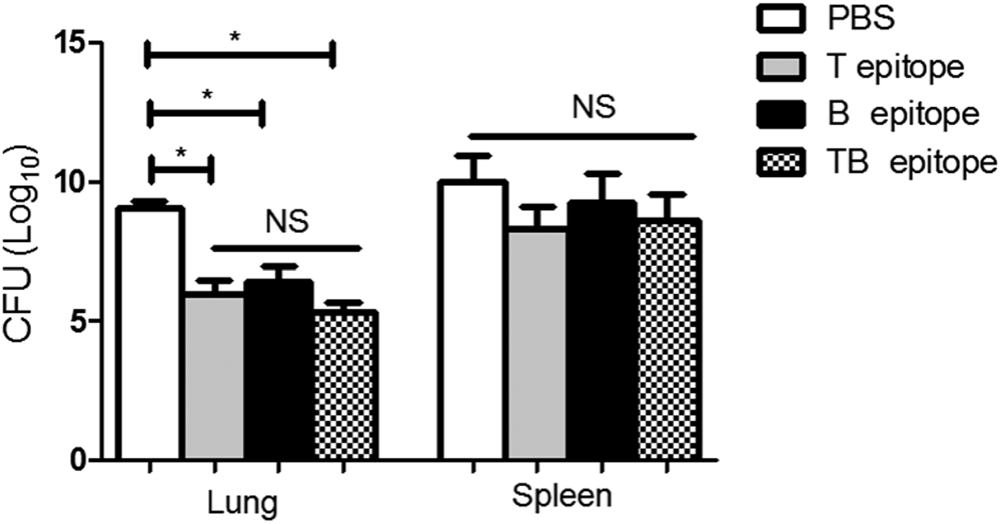
The bacteria load in the immunized mice. Thirty days after the last immunization, mice were challenged with 5 × 10^5^ CFU *Brucella* for 2 weeks. The lung and spleen cells were isolated and incubated in *Brucella* agar for 3 days at 37 °C with 5% CO2. Bacteria loads were calculated. Data were obtained from five mice of two independent experiments. Data are shown as mean ± SD. One-way ANOVA was used for analysis. **P < *0*.*01*. *P < *0*.*05*.

To determine whether the T epitope, B epitope or TB epitope had a protective effect against *B*. *melitensis* infection, the immunized mice were challenged with *B*. *melitensis*. Then the lung and spleen cells were isolated for the bacterial culture. Compared with the control group in lung, the bacterial loads of the immunized mice were significantly lower (*P* < *0*.*05*) (Fig. 3) and there is no significant difference between T epitope, B epitope or TB epitope immunized groups (*P > 0*.*05*). However, when compared with the bacterial load in spleen, there was no significant difference between these groups. These results indicate that T epitope, B epitope or TB epitope immunization could protect mice from *B*. *melitensis* infection in lung but cannot confer protection against its dissemination.

## Discussion

Here, in this study, we first demonstrated that OMP31 peptides were of high immunogenicity, which could induce both humoral immunity and Th1 cell response dramatically. Furthermore, we observed that OMP31 peptides could protect the host from *B*. *melitensis* infection.

OMP31 contains both B cell and T cell epitopes. To further study the immunogenicity of the peptide, we synthesized peptides containing T epitopes, B epitopes or TB epitopes and then immunized mice intranasally. We found that the levels of IgG1 and IgG2a in serum of B cell epitope and TB epitope immunized mice were higher than those in control group. And, IgG2a/IgG1 ratio in the immunized mice was higher. Specific antibodies have been used as important indicators for evaluating candidate vaccines^31^. Clausse, M. *et al*. have synthesized rBLSOmp31 and found the protein could induce IgG2a and IgG1 responses in the immunized mice^32^. Similarly, *Brucella* Omp2b protein is found to induce humoral immune responses and the production of IgG2a^33^. In this study, we found that *Brucella* peptides could also induce the humoral immune response of the body. Since *Brucella* infections can be acquired through mucosal membranes including respiratory tract, we would like to know whether peptides can induce the local mucosal immune response through intranasal administration. The results demonstrated that peptides containing B cell epitope and TB epitope could elicit the mucosal sIgA response. sIgA represents the hallmark of mucosal immune response. Moreover, sIgA also protect the host by binding to the surface of luminal microbes and toxins to prevent them from attaching to epithelial cells^34^. Therefore, vaccination with peptides containing B cell epitope and TB epitope through intranasal administration induced the antibody immune response and mucosal immunity of respiratory tract.

We found that the humoral immune response in mice immunized with B and TB epitopes was dominated by the IgG2a subtype. Studies have reported that IgG2a is mainly associated with Th1 profiles, and resistance to *Brucella* depends on enhanced Th1 immunity^35,36^. In this study, we also tested if Omp31 peptide could induce the Th1 immune response in mice by using IFN-γ ELISPOT. The lymphocytes in the respiratory draining lymph nodes and spleen from T epitope and TB epitope immunized mouse were stimulated by heat-killed RB51 *in vitro*. We found that the number of IFN-γ producing T cells was significantly increased, which indicates that peptides containing T epitope and TB epitope could efficiently induce Th1 immune response. Recent studies have used *Brucella* antigen Omp2b^37^ and Omp31^23^ to develop vaccines for *Brucella* and found that Th1 immune response could be induced. However, these candidate vaccines were not peptide-based vaccines. Afley, P. *et al*. have used a synthesized T cell polypeptide to induce the Th1 immune response^38^. Similarly in this study, we found that the peptides containing T epitope and TB epitope could activate IFN-γ producing T cells in respiratory draining lymph nodes and spleen. As such, the data presented here, argue that the peptides containing T epitope and TB epitope of Omp31 could induce the Th1 immune response. Importantly, for the first time, we identified the TB epitope of Omp31 and found that it could induce both humoral and Th1 immune responses.

To evaluate the protective capacity of peptides containing T cell epitope, B cell epitope and TB epitope, we used a mouse model challenged with *B*. *melitensis*. We found that bacterial loads in the lungs of the T cell epitope, B cell epitope and TB epitope immunized mice were significantly lower, indicating that T cell epitope, B cell epitope and TB epitope immunization could provide partial protection against *Brucella* infection in lung. However, this was not observed in the spleen. We speculate that this difference may be related to the different immune responses between the lung (respiratory tract) and the spleen. Studies have found that the immune response of respiratory tract in mice not only enhances T cell immune responses in murine pulmonary mucosa and the whole body, but also promotes the secretion of more specific sIgA in the respiratory tract mucosa^39,40^. In addition, we found that sIgA level in the bronchoalveolar lavage fluid was increased after immunization with peptides containing T epitope, B epitope and TB epitope. Therefore, we suppose that in the lungs, not only the cellular immune response is activated, but also sIgA is produced. However, in the spleen, there is no such response. In summary, our results demonstrate that peptides containing T epitope, B epitope and TB epitope have strong immunogenicity and can induce both Th1 immune response and humoral immune response, thus providing protection against *B*. *melitensis* infection in lung. These data presented in this study have important ramifications for the development of effective mucosal *Brucella* vaccines in future.

## Materials and Methods

### Animals

BALB/c mice (6 weeks old, female, 10 for each group) were purchased from the animal center of Xinjiang Medical University (Urumqi, Xinjiang, China). Mice were kept in standard condition with free access to food and water. All animal experiments were conducted according to the ethical guidelines of State Key Laboratory Incubation Base of Xinjiang Major Diseases Research All animal experiments in this study were performed in accordance with the Guidelines for Animal Experimentation and were approved by the Animal Research Committee of XinJiang Medical University (approval number A-20130216-155).

### Epitope prediction and analysis

We predicted the T cell epitope of OMP31 with SYFPEITHI (http://syfpeithi.de/) and ProPred Prediction Server (http://www.imtech.res.in/raghava/propred1/). The B cell epitope of OMP31 was predicted using DNAstar (USA lynnon biosoft) and IEDB software (http://tools.immuneepitope.org/bcell). The predicted T cell, B cell epitopes and TB epitopes of OMP31 (Table 1) were synthesized by Shanghai Qiangyao Biotechnology (Shanghai, China). The amino acid sequences shared by T cell epitopes and B cell epitopes were defined as TB epitopes.

**Table 1:** Sequences of epitopes used in this study.

Epitope	Residues	Sequence	Synthetic peptides
T	162-169 202-223	KSAFNLGD KSEYLYTDLGKRNLVD VDNSFL	KSAFNLGD KSEYLYTDLGKRNLVDVDNSFL- amide
B	164-179 211-226	AFNLGDDASALHTWSD GKRNLVDVDNSFLESK	AFNLGDDASALHTWSDGKRNLVDVDNSFLESK-amice
TB	164-169 211-223	AFNLGD GKRNLVDVDNSFL	AFNLGD GKRNLVDVDNSFL-amide

### Immunization and *B*. *melitensis* Challenge

**Figure 4:**
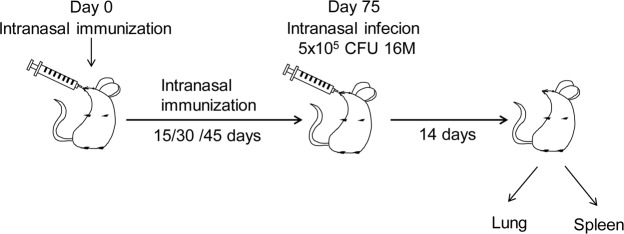
The process of immunization and *B*. *melitensis* infection. Mice were immunized intranasally (*i*.*n*, 15 μL per nostril) with PBS (Control group), 30 μg T epitopes (T epitope group), B epitopes (B epitope group) and TB epitopes (TB epitope group), respectively. Enhanced immunization was performed on the 15th, 30th and 45th day after the primary immunization, respectively. On the 75th day after the first immunization, the mice were intranasally infected with 5 × 10^5^ CFU *Brucella* strain 16 M. Fourteen days after nasal infection, mice were sacrificed after anesthesia. The lung and spleen of mice were isolated for subsequent studies.

The process of immunization and *B*. *melitensis* challenge was shown in Fig. 4. BALB/c mice were randomly divided into four groups, including control group, T group, B group, TB group. Mice were immunized intranasally (*i*.*n*, 15 μL per nostril) with PBS (Control group), 30 μg T epitopes (T epitope group), B epitopes (B epitope group) and TB epitopes (TB epitope group), respectively, for 0, 15, 30, and 45 days. All animal experiments with live *B*. *melitensis* were performed in BASL-3 facility. To assess the protective effect of T cell epitopes, B cell epitopes and TB epitopes, mice were infected with 5 × 10^5^ CFU *B*. *melitensis* (strain 16 M) by *i*.*n*. infection at 30 days after the last immunization. At 14 days post infection, all mice were sacrificed by cervical dislocation after inhalation of isoflurane. The respiratory lymph nodes (head, neck, and lower respiratory lymph node) and spleen was removed under sterile conditions and the homogenate was prepared for cell isolation. The trachea was cannulated, and the lungs were gently lavaged 3 times with 1 mL of sterile PBS to obtain bronchoalveolar lavage fluid. Blood was collected from the retro-orbital sinus using a Pasteur capillary pipette without anticoagulant and placed in coagulation microtubes. After centrifugation, serum was collected.

### ELISA

To detect the immunogenicity of T cell epitope, B cell epitope and TB epitope of OMP31. Antigen-specific IgG1 and IgG2a titers in the immunized mice were tested by ELISA. Briefly, the T epitope, B epitope and TB epitope (1 μg/ml) were coated on 96-well polystyrene plate (Greinerbio-one, Frickenhausen, Germany) at 100 μL/well and incubated at 4 °C overnight. The plate was rinsed with TBS-T (Tris-buffered saline, pH 7.4, containing 0.05% Tween-20) for 4 times, followed by blocking with PBS containing 10% fetal bovine serum (FBS) at 37 °C for 2 h. Then, 100 μl of mouse serum diluted at 1:100 was added to the well and incubated at room temperature for 2 h. After rinsing, horseradish peroxidase conjugated goat-anti-mouse IgG1 or IgG2a antibodies (BD Pharmingen, Santiago, USA) were added and then incubated at 37 °C for 1 h. Substrate solution (BD OptEiA, BD Pharmingen, Santiago, USA) were added to each well. After 10 min of incubation at room temperature, 20% H_2_SO_4_ was added to terminate the reaction. The absorbance value was measured using a microplate reader (Bio-Tropsch Tek Instruments, Winooski, Vt., USA) at a wavelength of 405 nm.

For measurement of sIgA, 96-well plates were coated with 0.25 g/well peptide (T epitope, B epitope and TB epitope) overnight at 4 °C. The bronchoalveolar lavage fluid (1:5 dilution) were added in triplicate into wells of the plates and incubated for 1 h at 37 °C. After washing, horseradish peroxidase conjugated goat anti-mouse IgA (1: 1,000 dilutions; KPL) was added and incubated for 1 h. Then reactions were completed by addition of the substrate as described above.

### Preparation and culture of mouse respiratory lymph node cell and splenocytes

Respiratory lymph nodes (head, neck, and lower respiratory lymph node) and spleens were harvested, cut into small pieces. Single-cell suspensions were prepared by mechanically dispersing the tissues through 70-μm cell strainers (Falcon BD Labware) and the cells were suspended in 10 mL PBS containing 5 mM EDTA. The mononuclear cells were isolated by Ficoll-Paque density gradient centrifugation. Then, the mononuclear cells were cultured in RPMI-1640 medium (RPMI-1640 supplemented with 2mM l-glutamine, 100 U/mL penicillin, 100 g/mL streptomycin and 10% heat inactivated FBS) at 37 °C, 5% CO_2_.

### IFN-γ ELISPOT

ELISPOT plates were coated with anti-mouse IFN-γ capture antibody (BD PharMigen, San Diego, CA) diluted 1:60 in PBS at 4 °C, overnight. The plate was then washed four times with PBST and once with PBS. Plates were blocked with 100 μL of RPMI-1640 medium containing 10% fetal calf serum for 1 h at 37 °C. The respiratory lymph nodes cells and splenocytes were added at a final concentration of 1 × 105/well in a final volume of 100 μL. Cells were stimulated with the heat-killed RB51 (10 μg/mL) (a source of *Brucella* protein without LPS), synthetic Toxoplasma SAG4 peptide (10 μg/mL). ConA (10 μg/mL) at 50 μL/well and PBS at 50 μL/well was used as the positive and negative controls, respectively. Then the plates were incubated at 37 °C with 5% CO2 for 24 h. After washing, 50 μL of biotin-labeled secondary antibody (BD PharMigen, San Diego, CA) was added and the plate was incubated at room temperature for 2 h. Then, the plates were washed with PBST and 50 μL streptavidin alkaline phosphatase (Amersham Life Science, Australia) was added and incubated at room temperature for 2 h. The spots were developed by adding BCIP/NBT developer (Moss, Inc, Pasadena, MD, USA) to each well for incubation for 40 min at room temperature. At last, plates were washed in water to stop reaction and the spots were counted using an ELISPOT Bio Reader-4000 (BIOSYS, GmbH, Germany).

### Analysis of *Brucella* CFUs

Lung and spleen were suspended in 1 ml of 0.9% normal saline containing 0.1% Triton X-100 and mechanically homogenized in tissue grinders. A total of 20 μl of undiluted homogenates and serial 10-fold dilutions of homogenates were inoculated on tryptic soy agar plates and incubated for 3–4 days at 37 °C in 5% CO_2_. *Brucella* CFUs were enumerated. The results were presented as the mean log_10_ CFU ± SD per group.

### Statistics

The data were analyzed by SPSS 13.0. When the data were normal distribution, they were shown as mean ± SD. In this study, one-way ANOVA was used for analysis in experiments with more than two groups. And student t test was used when there were two groups in experiments. When the data was non-normal distribution, rank sum test was used. A *P* < *0*.*05* was considered statistically significant.
